# The effects of filtration and centrifugation on the gut microbiota in fecal microbiota transplantation preparation

**DOI:** 10.3389/fmicb.2026.1768356

**Published:** 2026-05-12

**Authors:** Yuchi Liu, Jinhua Gong, Wenhua Wang, Xinyue Li, Huijie Jia, Ruixue Wang, Qiqi Sun, Ru Zhang, Yuying Zhang, Liuye Huang

**Affiliations:** 1 Qingdao Medical College, Qingdao University, Qingdao, China; 2 Department of Gastroenterology, The Affiliated Yantai Yuhuangding Hospital of Qingdao University, Yantai, China; 3 Department of Gastroenterology, Weifang People’s Hospital, Shandong Second Medical University, Weifang, China

**Keywords:** 16 s rDNA sequencing, centrifugation, fecal microbiota suspension, fecal microbiota transplantation, filtration, human feces, microbial viability

## Abstract

**Background:**

The preparation method of fecal microbiota suspensions is crucial for reliable fecal microbiota transplantation research. However, current protocols vary significantly in processing parameters, potentially compromising the comparability of studies. Systematic comparisons of how different preparation techniques affect the microbial community are still lacking.

**Methods:**

Fresh fecal samples from nine adult volunteers were processed via filtration and centrifugation at various speeds. Microbial viability was assessed via live/dead staining and colony forming unit enumeration. 16S rDNA sequencing was performed to analyze bacterial diversity and taxonomic composition.

**Results:**

The microbial composition and functional potential did not differ between the filtration alone group and the fresh fecal group, and filtration resulted in the lowest bacterial mortality. Mortality increased with increasing centrifugation speed. Centrifugation selectively affected the abundance of some genera (e.g., *Delftia* and *Acinetobacter*). High-speed supernatants presented markedly lower OD_600_ values than low-speed supernatants did, and differential centrifugation visibly reduced the amount of debris.

**Conclusion:**

Filtration alone best preserved fecal microbial viability, composition and functional potential. Centrifugation enrichment becomes selective at high speeds. Differential centrifugation offered superior impurity removal. The preparation strategy should be tailored to the research or therapeutic goal.

## 1 Introduction

The human gut microbiota, often referred to as the “second genome,” actively participates in metabolic regulation, immune system modulation, and pathogen defense (Tuddenham and Sears, 2015). These functions have propelled fecal microbiota transplantation (FMT) into the spotlight. FMT transfers processed feces from healthy donors to patient intestines, aiming to rebuild beneficial microbial communities for disease treatment (Borody and Khoruts, 2011; Quaranta et al., 2022). FMT is a key tool for rebuilding the gut ecosystem. FMT has demonstrated efficacy in treating recurrent *Clostridioides difficile* infection (Rokkas et al., 2019), inflammatory bowel disease (Zhao et al., 2020), metabolic syndrome (Proença et al., 2020; Agus et al., 2021), and other disorders (Zhao et al., 2021; Fan et al., 2025a). The clinical success of FMT has promoted research on the functional mechanisms and therapeutic potential of the gut microbiota.

However, the effectiveness of FMT is variable (Danne et al., 2021; Waller et al., 2022; Karimi et al., 2024; Basson et al., 2020). The variability in FMT efficacy is attributed to differences in clinical indications, administration routes, treatment frequencies, fecal preparations, and donors (Zhang et al., 2023). Its production is a key determinant of its therapeutic effect (Cui et al., 2016; Zhang et al., 2020). The preparation of a fecal suspension includes sample collection, weighing, homogenization, filtration, centrifugation, concentration measurement, cryopreservation and freeze-drying (Fan et al., 2025a; Bénard et al., 2023), which involve multiple factors, such as whether the sample is operated in an anaerobic environment, fresh or frozen, and time. Current studies have focused on the effects of anaerobic operation (Bellali et al., 2019), the use of fresh or frozen feces (Nicco et al., 2020), and operation and time (Ott et al., 2004) on fecal bacterial suspensions. Although anaerobic processing theoretically better preserves obligate anaerobes (Bellali et al., 2019), fecal microbiota transplantation preparation is frequently conducted under ambient atmospheric conditions in current clinical practice (Yadegar et al., 2024). Furthermore, few studies have been conducted on the potential impacts of filtration (pore size selection) and centrifugation (speed) on microbial viability and composition. Approximately one-third of laboratory studies do not specify filtration or centrifugation steps (Secombe et al., 2021), while variations exist in fecal processing methods among researchers conducting detailed FMT protocols. Lin et al. and Randolph et al. prepared bacterial suspensions for FMT via filtration only to remove large debris. Moreover, different researchers have employed various filtering materials with different pore sizes, such as glass wool (Lin et al., 2019), gauze (Randolph et al., 2025a), 2 mm × 2 mm sterile sieves (Zain et al., 2022), and 70 μm filters (Wu et al., 2021). Most protocols now combine filtration with centrifugation (Zain et al., 2022; Hamilton et al., 2012), but centrifugation methods and speeds also vary considerably, including various single-step centrifugation protocols at different speeds and durations [such as 500 × g for 5 min (Stebegg et al., 2019), 800 × g for 3 min (Lai et al., 2018), 1,000 × g for 3 min (Zhao et al., 2021), 2,000 × g for 5 min (Liu et al., 2020), 3,000 × g for 10 min (Randolph et al., 2025a), 5,000 × g for 20 min (Zhou et al., 2019; Randolph et al., 2025a) and 6,000 × g for 15 min (Perez et al., 2016; Hu et al., 2018)], as well as differential centrifugation strategies with different speed and time combinations [such as 400 × g for 10 min followed by 3,000 × g for 25 min (Zain et al., 2022) or 400 × g for 5 min followed by 6,000 × g for 10 min (Sedeek et al., 2025)]. Mingaila et al. identified centrifugation speed as a critically understandardized variable, with speeds ranging from 500 × g to 16,000 × g lacking empirical rationale (Mingaila et al., 2023). The study by Randolph et al. showed that double centrifugation can remove impurities and boost bacterial concentrations in animal models (Randolph et al., 2025b). Because of the differences in the preparation process of fecal suspensions among different researchers, it becomes more difficult to compare independent studies (Danne et al., 2021).

With respect to the impact of filtration and centrifugation on the fecal microbiota suspension used for FMT, Mi et al. (2023) used 16S rDNA sequencing to evaluate microbial feces and demonstrated that stirring followed by gauze filtration preserved a community structure most similar to that of the original feces, whereas centrifugation significantly altered the composition. Existing studies have found that increased centrifugation speed affects bacterial activity. The study attributes the velocity-dependent mortality to intensified shear stress and compressive forces. They damage the outer membrane of gram-negative bacteria or the peptidoglycan wall of gram-positive organisms (Peterson et al., 2012). Regarding filtration, the pore size and material of the filtration medium can directly affect bacterial structural integrity. Membranes with excessively small pores or rough surfaces are more likely to cause bacterial cell lysis (Gasol and Morán, 1999).

Our study, which uses large-volume human fecal samples, combines live/dead staining for viability assessment and 16S rDNA profiling for diversity analysis to evaluate the effects of filtration and different centrifugation parameters. These findings are expected to provide evidence for preclinical FMT studies, thereby enhancing the controllability and therapeutic consistency of FMT.

## 2 Materials and methods

### 2.1 Volunteer recruitment and fecal sample collection

This was an experimental laboratory study comparing different processing methods for human fecal samples. The samples were collected in Yantai, Shandong Province, China. Sample collection and processing were conducted from 10 to 12 August 2025. The participants were required to have no history of antibiotic use in the past 3 months. Participants were excluded if they had used antibiotics, probiotics, or proton pump inhibitors within the past 3 months. All participants were informed of the experiment and signed an informed consent form. The protocol was approved by the Ethics Review Committee of The Affiliated Yantai Yuhuangding Hospital of Qingdao University [2025–665]. All laboratory procedures were performed at the Central Laboratory, Yantai Yuhuangding Hospital. No formal sample size calculation was performed because of the exploratory nature of this method optimization study. Nine healthy adult volunteers (3 males, 6 females; mean age 31.2 ± 11.1 years) were recruited as independent biological replicates (*n* = 9). All donors underwent physical examination and laboratory screening including complete blood count, liver and renal function tests. Exclusion criteria included antibiotic, probiotic, or proton pump inhibitor use within 3 months; gastrointestinal illness (diarrhea, constipation, abdominal pain) or fever within 3 months; recent foreign travel; or major dietary changes (e.g., initiation of vegan diet, high-protein diets, alcohol binge drinking) within 1 week prior to donation. For each donor, fecal samples were aliquoted and processed under different conditions (filtration alone, various centrifugation speeds, etc.). In this study, fresh fecal samples were collected from nine adult volunteers via sterile sampling bags (A38254, Beckman Coulter, Brea, CA, United States). All the samples were immediately stored in iceboxes (maintained at 2–8 °C), transported to the laboratory within 30 min and processed without delay.

### 2.2 Fecal suspension preparation and grouping

Fresh fecal samples were designated the fresh fecal group (FS group). After being weighed, the samples were subjected to 1 × PBS dilution (1 g feces: 5 mL PBS, w/v) followed by 2 min of manual homogenization in sterile homogenizing bags (130,209,014, Beckman Coulter, Brea, CA, USA) at approximately 60–80 shakes per minute until the suspension appeared uniform without visible particulate clumps (>2 mm). The filtrate obtained by homogenizing bag filtration was sequentially filtered at room temperature through vacuum-driven filter units (4524-G300500C, POMEX, China) containing 100 μm and subsequently 30 μm sterile nylon mesh filters (Delvstlab, China) to obtain the fine filtration group (FF group). The FF group represents the microbial suspension passing through the 30 μm filter. Due to high inter-donor variation in fecal consistency, the FF filtrate was standardized using an iterative dilution-verification method. An aliquot (1 mL) of the original FF filtrate was diluted 100-fold (10^−2^) for spectrophotometric estimation of optical density at 600 nm (OD_600_) using a Biowave II spectrophotometer (Biochrom, England). Because the original filtrate frequently exceeded the linear detection range, a fixed proportional calculation was not feasible. Instead, based on this initial reading (e.g., 0.53), the remaining bulk filtrate was empirically diluted with sterile PBS. Subsequently, another aliquot (1 mL) was taken from this diluted filtrate and subjected to 10^−2^ dilution, and the OD_600_ was measured again. This process was iterated until the 10^−2^ dilution of the adjusted filtrate yielded OD_600_ = 0.4. For centrifugal separation, 30 mL filtered fecal suspensions of the adjusted suspension were processed in 50 mL centrifuge tubes under various conditions: (1) FC group - supernatant collected after centrifugation at 500 × g for 5 min; (2) FLC, FMC, and FHC groups - bacterial pellets collected after centrifugation at 4,000 × g, 6,000 × g, and 10,000 × g for 15 min, respectively; (3) FPMC and FPHC groups - supernatants from 500 × g centrifugation were further centrifuged at 6,000 × g or 10,000 × g for 15 min to collect bacterial pellets. All the above centrifugation treatments were performed in a 4 °C prechilled centrifuge (75,004,380, Thermo Fisher Scientific, United States). Live/dead bacterial staining and 16S rDNA sequencing were conducted on the collected samples. All procedures were conducted in a Biosafety Level 2 cabinet with appropriate personal protective equipment to minimize risks.

**Figure 1:**
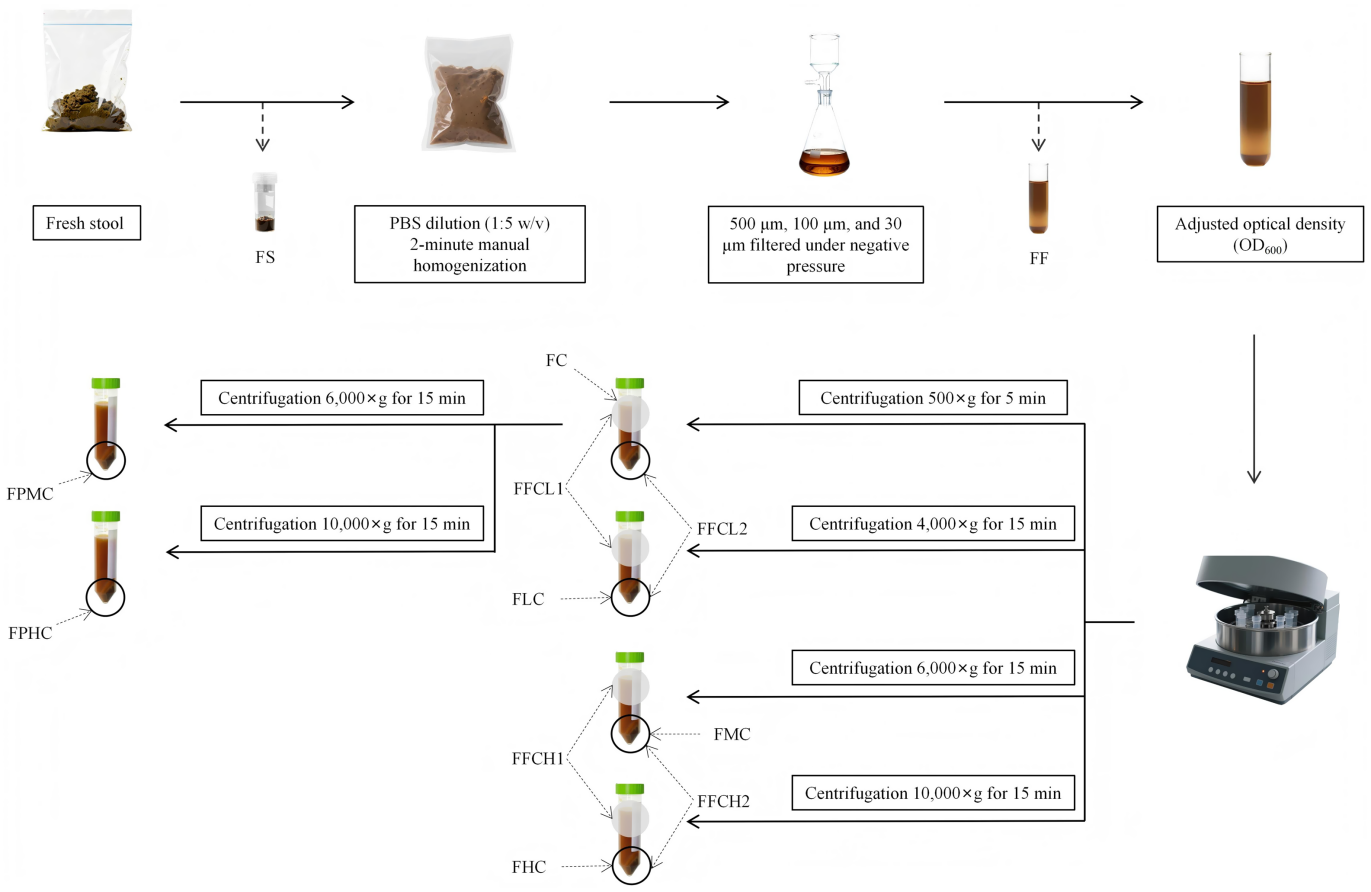
Flowchart of the experimental design.

To assess potential microbial loss, we sampled both the supernatant and pellet from the centrifugation groups and designed corresponding subgroups: (1) FFCH (FMC + FHC), representing high-speed centrifugation, where FFCH1 denotes the pooled supernatants from FMC and FHC groups, and FFCH2 denotes the pooled pellets from FMC and FHC groups; and (2) FFCL (FC + FLC), representing medium-low-speed centrifugation, where FFCL1 denotes the pooled supernatants from FC and FLC groups, and FFCL2 denotes the pooled pellets from FC and FLC groups. Both the supernatants (FFCH1, FFCL1) and pellets (FFCH2, FFCL2) from these combined groups were analyzed for microbial diversity and taxonomic composition differences. The OD_600_ values of all the supernatants were measured to quantify potential microbial loss. The workflow of the experimental design is shown in Figure 1.

### 2.3 Evaluation of microbiota viability

Microbial viability was evaluated via the LIVE/DEAD Bacterial Staining Kit (DMAO/PI, Beyotime® C2030S). The FF group and all centrifugation pellet groups (FLC, FMC, FHC, FPMC, FPHC) were washed with sterile 1 × PBS and adjusted to OD_670_ ≈ 0.3. The samples were stained with a 1:100 (v/v) ratio of the working solution (prepared by diluting 1,000 × DMAO and PI stocks in detection buffer) and incubated at 37 °C for 15 min in darkness. DMAO (ex/em: 503/530 nm) labeled all bacteria (live/dead) with green fluorescence, whereas PI (ex/em: 535/617 nm) penetrated only membrane-compromised bacteria (dead), emitting red fluorescence. Dual-channel fluorescence images were captured via a Zeiss Observer 7 microscope (20 × objective), with five random fields per sample analyzed via Zen 2011 Blue software. Live bacteria exhibited green fluorescence, whereas dead bacteria presented dual green/red signals (merged yellow). The estimated proportion of dead particles was calculated as the percentage of PI-positive cells relative to total DMAO-positive cells. For LIVE/DEAD staining, three technical replicates were performed per donor per treatment group to account for measurement variability. This method sensitively and quantitatively assessed microbial viability across processing groups.

To validate membrane integrity assessments with functional viability measures, colony forming unit (CFU) enumerations were performed. For CFU enumeration, processed samples were resuspended in sterile PBS and adjusted to OD_600_ = 0.3 to standardize the cell density across all groups. Serial dilutions (10^−2^, 10^−3^, and 10^−4^) were prepared from these standardized suspensions, and 100 μL were plated onto BHI agar in triplicate. The plates were incubated aerobically at 37 °C for 48 h. Colonies were counted and expressed as CFU/mL of the standardized suspension (OD_600_ = 0.3). This standardization allowed direct comparison of bacterial viability across processing methods at equivalent cell density. Three biological replicates (donors) were analyzed.

### 2.4 Sequencing and bioinformatic analyses

For pellet groups (FLC, FMC, FHC, FPMC, FPHC), the entire pellet was scraped and resuspended in sterile PBS. For the supernatant groups (FC), 5 mL of the supernatant was collected after gentle mixing. For filtration groups (FF), 5 mL of the original filtrate was collected. All samples were immediately snap-frozen in liquid nitrogen and stored at −80 °C until DNA extraction(Yang et al., 2020). Fecal microbial DNA was extracted via a QIAamp DNA Fecal Mini Kit (Qiagen, Hilden, Germany). PCR amplification was carried out via an ABI 2720 Thermal Cycler (Thermo Fisher Scientific, United States). We used Multiskan™ GO spectrophotometry (Thermo Fisher Scientific, United States) to quantify bacterial genomic DNA as the template for amplification of the V3-V4 hypervariable region of the 16S rDNA gene in three replicate reactions with forward (Illumina adapter sequence 5’-CCTACGGGNBGCASCAG-3’) and reverse (Illumina adapter sequence 5’-GGACTACNVGGGTWTCTAAT-3’) primers. Replicate PCR products were pooled and purified with Agencourt AMPure XP magnetic beads (Beckman Coulter, United States). A TopTaq DNA Polymerase Kit (TransGen, China) was used. The purity and concentration of sample DNA were assessed via a NanoDrop 2000 Spectrophotometer (Thermo Fisher Scientific, United States). Paired-end sequencing was performed by Treatgut Biotechnology Co., Ltd., with a HiSeq 2,500 (Illumina, San Diego, CA, United States) with PE 250 bp reagents.

After sequencing, the raw paired-end reads were assembled via flash (Magoč and Salzberg, 2011). The primers were removed via Cutadapt (Martin, 2011), and the clean tags were obtained by removing the lower reads via Cutadapt (Martin, 2011). Chimera checking and amplicon sequence variant (ASV) clustering were performed with the clean tags via DADA2 in QIIME2 following the pipeline. In detail, all reads were denoised using DADA2 to resolve single-nucleotide differences; then, chimera checking was performed via the consensus method. Representative sequences were generated, and a final ASV table was created. The representative sequences of the ASVs were aligned against the silva_132_97_16S (DeSantis et al., 2006) database for taxonomic classification via the RDP (Cole et al., 2014) Classifier. The phylogenetic trees were produced via FastTree (Price et al., 2009).

### 2.5 Functional profiling on the basis of bacterial taxonomy

To predict metagenomic function content, the Phylogenetic Investigation of Communities by Reconstruction of Unobserved States (PICRUSt) (Langille et al., 2013) was used to predict which genes were present via 16S data. The software used a computational approach to predict the functional pathway from the 16S rDNA reads. First, the reads were compared against a reference collection, the GreenGenes (DeSantis et al., 2006) database, May 2013 version, and the closed-reference ASV table was built via QIIME (Caporaso et al., 2010). The resulting ASV table was normalized by normalize_by_copy_number.py, and metagenome predictions were conducted via predict_metagenomes.py. Significant differences were determined via ANOVA. The results were visualized via custom R script based on ggplot2 (Wickham, 2016).

### 2.6 Statistical analyses and visualization

The estimates of alpha diversity were based on an evenly rarefied ASV abundance matrix and included observed richness. The species, Shannon, Simpson, ACE, Chao1 and Pielou’s evenness (J’) indices were calculated via the R package vegan (Oksanen et al., 2013). The significance of the differences in the measured *α* diversity metrics across samples was tested via a nonparametric Kruskal–Wallis rank sum test and Benjamini–Hochberg correction. The *β*-diversity of the samples was measured via the Bray-Curtis distance on the basis of an evenly rarefied ASV abundance table. β-diversity could be used to estimate the difference in community structure between samples. Statistical differences in measured β-diversity metrics across groups were determined via PERMANOVA with 999 permutations via adonis in the R package vegan (Oksanen et al., 2013). Shared ASVs were calculated and visualized via the R package Venn Diagram (Chen and Boutros, 2011). The taxon abundance was measured and plotted via ggplot2 (Wickham, 2016). LEfSe (Paulson et al., 2013) analysis was performed to identify taxa with different abundances in the different groups. LEfSe (Paulson et al., 2013) is an algorithm for high-dimensional biomarker discovery that identifies genomic features characterizing the differences between two or more biological conditions. Moreover, indicator analysis on the basis of genera was conducted via the R package indicspecies (De Cáceres and Legendre, 2009). Indicator taxa analysis was a way to calculate the probability that any taxon is found in different groups. A taxon with a high indicator value had a high probability of being found within a given treatment and a low probability of being found outside the treatment, and the *p* values were corrected with the method of Benjamini–Hochbery via p.adjust in R. Finally, the results were visualized via the custom R script based on ggplot2 (Wickham, 2016). These analyses were performed via R v3.4.1.

The estimated proportion values of dead particles based on the Live/Dead staining experiment were averaged across three technical replicates for each biological replicate, and three biological replicats per group were analyzed in SPSS 26.0. After confirming normality (Shapiro–Wilk test, *p* > 0.05) and homogeneity of variance (Levene’s test, *p* > 0.05), group differences were evaluated via one-way ANOVA followed by Tukey’s *post hoc* test (*α* = 0.05). The results are presented as the means ± SD, and *p* < 0.05 was considered statistically significant.

## 3 Results

### 3.1 Clinical characteristics of the fecal donors

A total of nine eligible fecal donors, comprising 3 males and 6 females with a mean age of 31.2 ± 11.1 years, were included. All donors were screened strictly according to the inclusion criteria. The detailed baseline demographic data are provided in Supplementary Table S1.

### 3.2 Rarefaction curve analysis

To evaluate the adequacy of our sequencing depth, rarefaction curve analysis was performed on all the samples. The average sequencing depth was 85,432 ± 12,847 reads per sample (range: 68,234–102,456 reads). The curves for all the samples plateaued with similar patterns, indicating that the sequencing depth was sufficient to capture the majority of the microbial diversity present and that the samples were comparable. The high number of valid sequence reads (over 90% of the raw reads) further confirmed the reliability and quality of the sequencing data for downstream analyses (Supplementary Figure S1).

### 3.3 Filtration and centrifugation differentially impact microbial viability

**Figure 2:**
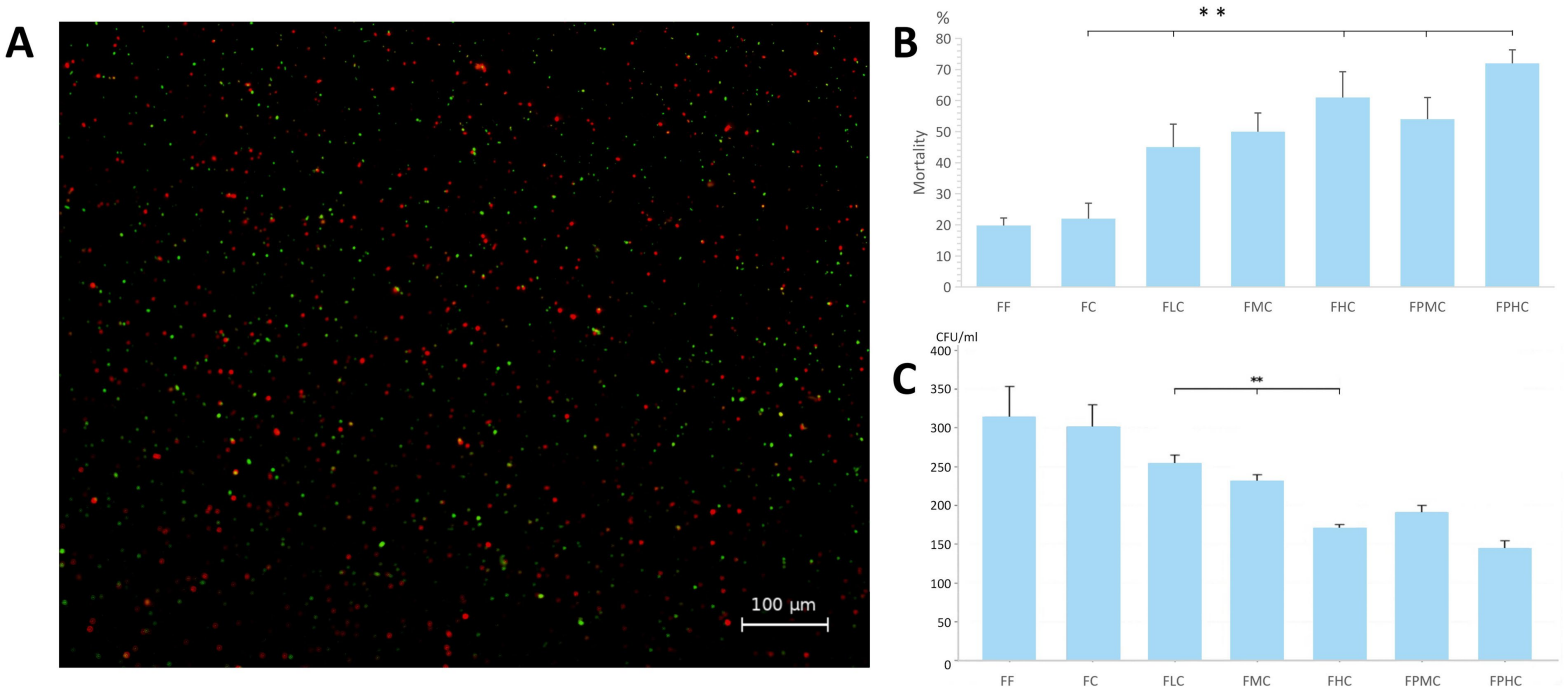
Assessment of fecal microbiota viability by LIVE/DEAD staining and CFU enumeration. **(A)** FPHC group microbial viability under a microscope. Live bacteria emitted green fluorescence, whereas dead bacteria displayed both green and red signals that merged into yellow. **(B)** Statistical analysis of the estimated proportion of dead particles. Compared with the FC and FF groups, all the centrifugation groups presented significant differences in mortality rates (*p* < 0.01). ** indicates *p* < 0.01 compared with the control group (one-way ANOVA followed by Tukey’s post hoc test). **(C)** Cultivable microbial viability assessed by CFU enumeration. ** indicates *p* < 0.01 (one-way ANOVA followed by Tukey’s post hoc test).

We assessed the impact of processing methods on microbial viability via live/dead staining. Under a fluorescence microscope, live bacteria emitted green fluorescence, whereas dead bacteria displayed both green and red signals that merged into yellow (Figure 2A). Staining validation with *E. coli* controls is shown in Supplementary Figure S2. The statistical analysis revealed that different treatment methods had effects on the estimated proportion of dead particles (Figure 2B). Compared with the FF group, all the centrifugal groups except the FC group presented significantly higher mortality rates (FLC: *p* = 0.003; FMC: *p* = 0.002; FHC: *p* = 0.001; FPMC: *p* = 0.001; FPHC: *p* = 0.001; Figure 2B). Furthermore, a trend toward increasing mortality was observed with increasing centrifugation speed. A significant difference in mortality was specifically noted between the FHC and PFHC groups (*p* = 0.04), but this was not observed in comparisons between the FMC and PFMC groups.

CFU enumeration corroborated LIVE/DEAD staining results (Figure 2C). FF and FC showed highest cultivable counts without significant difference. CFU decreased progressively with centrifugation speed (*p* < 0.05 between groups). The CFU counts in the differential centrifugation group were all lower than those in the simple centrifugation group at the same rotational speed.

### 3.4 Microbial community differences between pellets and supernatants

**Figure 3:**
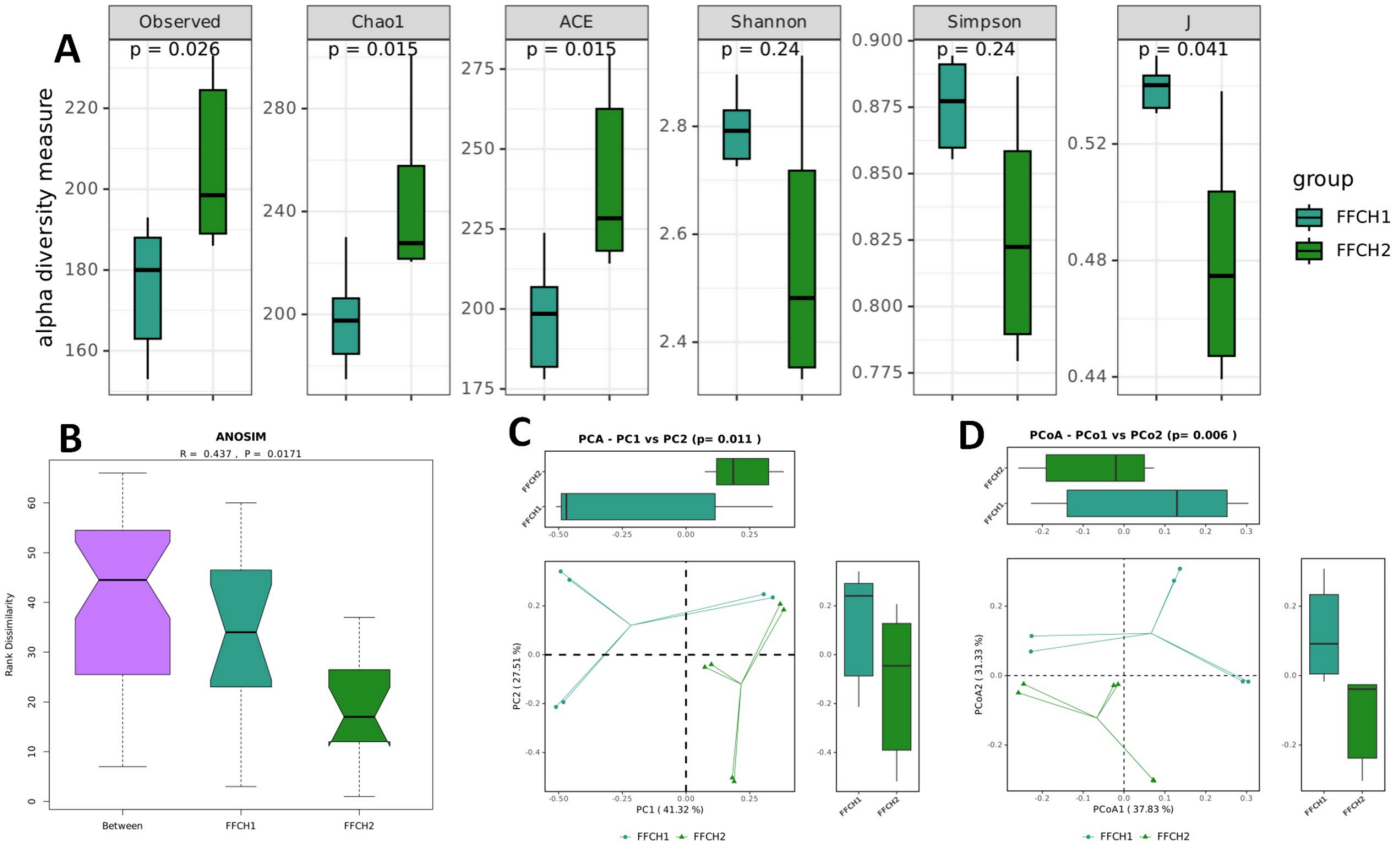
Comparison of microbial community alpha and beta diversity between the FFCH1 and FFCH2 groups. **(A)** Comparison of different alpha diversity indices between the two groups, showing significant differences in the observed, Chao1, ACE, and J indices (***p*** < 0.05). **(B)** ANOSIM results between the two groups, with R = 0.437 and *p* = 0.0171, indicating significant differences. **(C)** and D. The results of PCA-based and PCoA principal component analysis, demonstrating significant distribution differences between FFCH1 and FFCH2 in PC1 and PC2 (*p* = 0.011 and *p* = 0.006).

To assess microbial distribution patterns during centrifugation and evaluate microbial loss, we performed 16S rDNA sequencing not only on the collected bacterial pellets but also on the supernatants. The results revealed significant differences in both *α*-diversity and *β*-diversity (Observed, Chao1, ACE, and J indices, all *p* < 0.05) between the high-speed centrifugation group FFCH1 supernatants and the FFCH2 pellets (Figure 3A), whereas no significant differences in diversity were observed between the low-speed centrifugation group FFCL1 supernatants and the FFCL2 pellets (Supplementary Figure S3). ANOSIM analysis further confirmed significant differences in community structure between FFCH1 supernatants and FFCH2 pellets (R = 0.437, *p* = 0.0171; Figure 3B). Both principal component analysis (PCA) and principal coordinate analysis (PCoA) revealed clear separation of the two sample groups along the PC1 and the PC2 axes. In the PCA analysis, the distribution differences along both PC1 and PC2 were statistically significant (*p* = 0.011 and *p* = 0.006, respectively; Figure 3C), and consistent distribution patterns were observed in the PCoA (Figure 3D).

**Figure 4:**
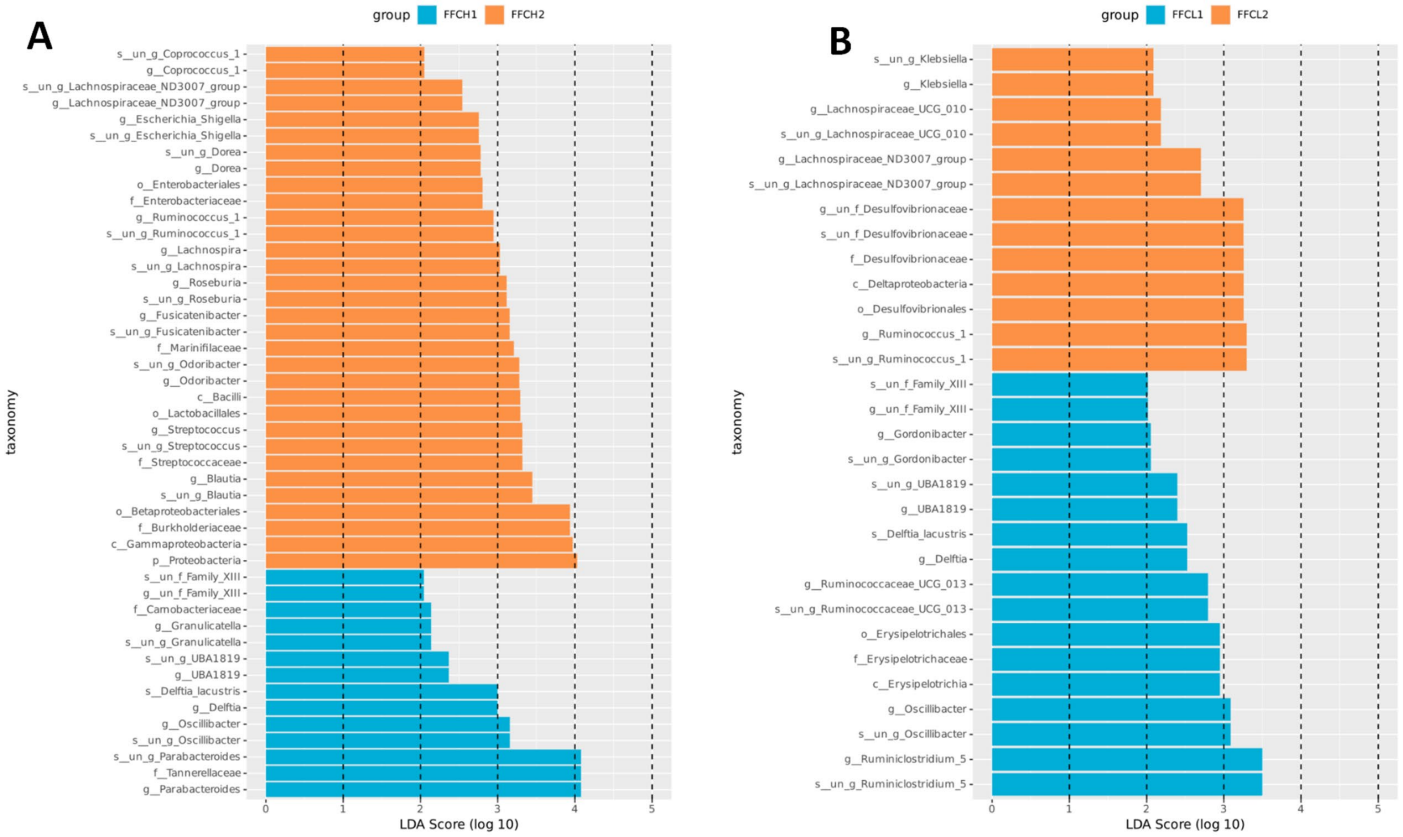
Discriminative taxa. (**A)** Discriminative taxa between the FFCH1 group and the FFCH2 groups. (**B)** Discriminative taxa between the FFCL1 group and the FFCL2 groups. By applying a linear discriminant analysis core threshold of 3, this method can effectively detect characteristic microbial species associated with specific groups.

The linear discriminant analysis (LDA) threshold was set as 3 to screen the characteristic microbiota of the corresponding groups. Fourteen kinds of characteristic differences in the microbiota were analyzed in the FFCH1 group, and 32 kinds of characteristic differences in the microbiota were analyzed in the FFCH2 group (Figure 4A). In the high-speed groups, taxa such as Granulicatella, UBA1819, Deftia, Oscillibacter, and Parabacteroides were enriched in the FFCH1 group, whereas Coprococcus_1, Lachnospiraceae_ND3007_group, Escherichia_Shigella, Dorea, Enterobacteriaceae, Ruminococcus_1, Lachnospiraceae, Roseburia, Fuscatibacter, Marinifilaceae, Odoribacter, Bacilli, Lactobacillales, Streptococcus, Blautia, Betaproteobacteriales, Burkholderiaceae, Gammaproteobacteria, and Proteobacteria were enriched in the FFCH2 group (Figure 4A). Similar partitioning was observed in the medium-low-speed groups, with distinct sets of taxa enriched in the FFCL1 supernatant (e.g., Family_XIII, Gordonibacter, UBA1819, Deftia, Ruminococcaceae_UCG_013, Erysipelotrichales, Oscillibacter, Ruminiclostridium_5) and FFCL2 pellet (e.g., Klebsiella, Lachnospiraceae_UCG_010, Lachnospiraceae_ND3007_group, Desulfovibrionaceae, Deltaproteobacteria, Ruminococcus_1; Figure 4B).

### 3.5 Microbial community structure across all processing groups

**Figure 5:**
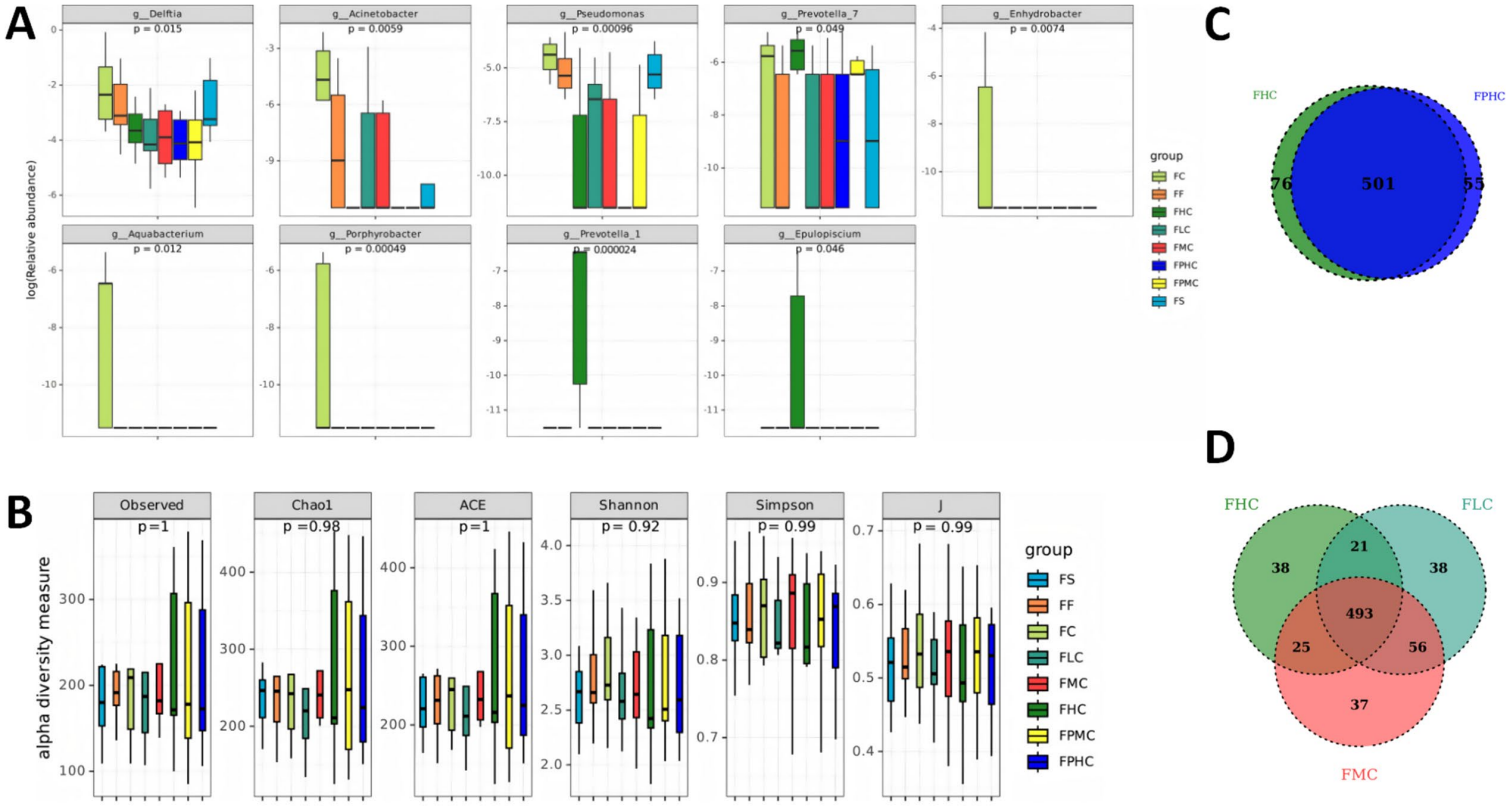
Microbial community composition and diversity in the different treatment groups. **(A)** The relative abundance of specific microbial taxa in different treatment groups. **(B)** Comparisons of different alpha diversity indices (observed, Chao1, ACE, Shannon, Simpson, J) among the groups, with no significant differences observed. **(C)** Venn diagram showing the differences in bacterial community structure between the FHC group and the PFHC group. **(D)** Venn diagram showing the differences in bacterial community structure among the FLC, FMC, and FHC groups.

We then broadened our analysis to compare the overall microbial community structure across all major processing groups. While the relative abundances of specific bacterial genera (e.g., g_*Delftia*, g_*Acinetobacter*, g_*Pseudomonas*, g_*Prevotella*_7, g_*Enhydrobacter*, g_*Aquabacterium*, g_*Peripherobacter*, g_*Prevotella*_2, and g_*Epulopiscium*) varied noticeably among the groups (Figure 5A), the overall *α* diversity (Figure 5B) and *β* diversity (Supplementary Figure S4A) were not significantly altered by any of the processing methods. This finding indicates that while specific populations may be enriched or depleted, the global diversity and structure of the community remain largely resilient to these manipulations. Furthermore, to assess the functional potential of the microbial communities under different processing methods, we performed functional prediction on the basis of 16S rDNA sequencing data via PICRUSt. While functional prediction analysis of KEGG metabolic pathways revealed no significant functional classification differences among the treatment groups, the COG functional classification prediction analysis identified several specific COG entries that exhibited significant differences. As shown in Supplementary Figure S4B, the computationally predicted functional gene categories showing differences included COG2189 (adenine-specific DNA methylase Mod), COG5008 (Tfp pilus assembly protein, ATPase PilU), COG5022 (myosin heavy chain), COG1139 (uncharacterized conserved protein containing a ferredoxin-like domain), COG3280 (maltooligosyl trehalose synthase), COG1282 (NAD/NADP transhydrogenase beta subunit), COG3472 (uncharacterized conserved protein), and COG3016 (uncharacterized iron-regulated protein). These results suggest that although the microbial community structure remained broadly similar across processing groups, the potential functional gene expression profiles may be specifically altered by the different preparation methods.

Comparative analysis of the Venn diagrams (Figures 5C,D) revealed that the sample processing methods had distinct influences on the microbial community structure. As illustrated in Figure 5C, the comparison between the FHC and FPHC groups revealed that the number of shared ASVs (501) overwhelmingly predominated, whereas the number of unique ASVs in each group was considerably lower. This finding indicates that differential centrifugation (FPHC) did not substantially alter the overall microbial structure compared with standard centrifugation (FHC). In contrast, the three-way comparison of the FHC, FLC, and FMC groups in Figure 5D demonstrated that, despite the presence of a large core microbiome (493 ASVs), each group separated by the centrifugation speed gradient retained a notable number of unique ASVs. This suggests that the centrifugation speed gradient (L, M, H) had a significantly greater effect on shaping the microbial community structure than did the differential centrifugation step.

### 3.6 OD_600_ of the supernatant discarded at different centrifugation speeds

As the centrifugation speed increased, the OD_600_ of the supernatant in the single-step centrifugation group significantly decreased. A similar decline was observed in the differential centrifugation group. When the maximum centrifugation speed was identical, the OD_600_ values of the supernatants did not differ significantly between the two protocols, indicating that the amount of bacteria retained in the supernatant was essentially the same. Even at 10,000 × g, the OD_600_ of the supernatant remained above 0.05, indicating that there were still a lot of microbes to throw away (Table 1).

**Table 1:** OD_600_ values of the supernatants.

Group	OD_600_			
	Mean	SD	95% CI	*p*
FLC	0.411	0.175	0.227–0.595	*p*<0.001
FMC	0.293	0.169	0.115–0.471	
FHC	0.139	0.101	0.033–0.245	
FPMC	0.280	0.190	0.081–0.479	
FPHC	0.133	0.086	0.042–0.223	

### 3.7 Impact of processing methods on impurity removal and macroscopic sediment stratification

**Figure 6:**
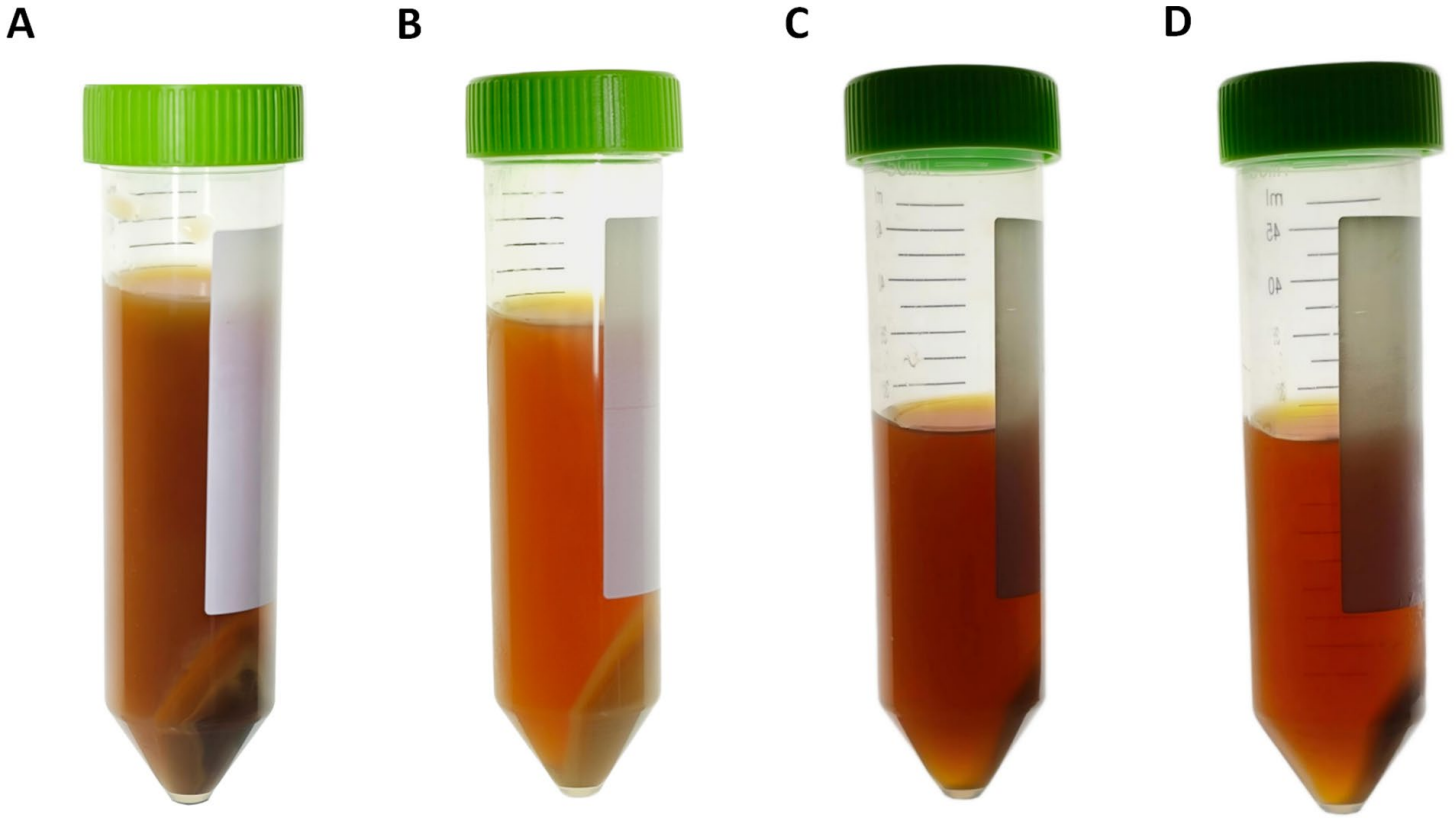
Macroscopic stratification of fecal suspensions following centrifugation. **(A)** Photograph of FHCs. **(B)** Photograph of the FPHC, which shares the same fresh feces as A. **(C)** Photograph of the FPMC. **(D)** Photograph of the FPHC, which shares the same fresh fecal material as C.

The pellets obtained from single-step centrifugation protocols displayed clear layering. They are characterized by an upper yellowish-brown layer and a lower darker brown layer with a firmer consistency (Figure 6A). In contrast, the pellets obtained from differential centrifugation lacked a distinct dark brown layer but instead consisted of a yellowish-brown layer (Figure 6B). Concurrently, the clarity of the supernatant increased markedly with increasing centrifugal force. This effect was most evident in the differential centrifugation groups (Figures 6C, 6D).

## 4 Discussion

In this systematic evaluation of the methods used to prepare the fecal microbiota suspensions, two principal findings emerged. First, filtration alone most effectively preserved microbial viability and functional potential, whereas centrifugation, particularly at higher forces, induced significant cell death and altered the predicted metagenomic profile. Second, centrifugation does not merely concentrate microbes uniformly. These centrifugal concentrations were selective to some extent. These findings have direct implications for standardizing FMT protocols, as the choice of processing method may influence the viability, composition, and potentially therapeutic efficacy of the transplanted microbiota.

CFU enumeration under aerobic culture conditions validated LIVE/DEAD staining trends. We acknowledge that aerobic culture conditions capture only aerotolerant, cultivable fractions. Consequently, our CFU data represent comparative trends in viability across processing groups. The centrifugation-induced physical damage affects bacterial cell envelopes universally regardless of oxygen requirements, supporting the validity of our comparative trends across processing methods. The superior performance of filtration in maintaining microbial viability is similar to prior observations in animal models (Mi et al., 2023). However, many structural differences exist between the rodent and human gut microbiota (Nguyen et al., 2015). It is important to extend these findings from animal to human fecal samples. The significant increase in bacterial mortality with centrifugal forces ≥ 4,000 × g is particularly noteworthy, as many existing protocols employ speeds within this range (Hamilton et al., 2012; Zain et al., 2022). In the present study, the mortality of microbiota collected from the supernatant at low centrifugal force (e.g., 500 × g) did not differ significantly from that of the filtration group, indicating that low-speed centrifugation exerts only a minimal effect on microbial viability. However, as the centrifugal force increased, mortality increased correspondingly. We attribute this to intensified shear stress and compressive forces that damage the outer membrane of gram-negative bacteria or the peptidoglycan wall of gram-positive organisms (Peterson et al., 2012). Centrifugation-induced shear stress and compressive forces causing cell envelope damage universally affect bacteria. We acknowledge that anaerobic cultivation would provide absolute viability values for strict anaerobes; however, our comparative trends across processing methods remain valid given the universal nature of centrifugation-induced physical damage. Notably, when the centrifugation force was further increased from 4,000 × g to 10,000 × g, bacterial mortality showed only an increasing trend without reaching statistical significance. We hypothesize that once the centrifugal force exceeds a critical threshold (approximately 500–4,000 × g in the present study), the increase in physical stress sufficient to disrupt bacterial membranes begins to plateau. Therefore, further increasing the centrifugation speed primarily enhances sedimentation efficiency rather than causing membrane damage.

The spatial distribution pattern of the microbiota during centrifugation also needs attention. In the present study, neither the *α*-diversity nor the *β*-diversity differed significantly between the supernatant and the pellet in the low-speed centrifugation groups. This finding implies that, at centrifugal forces ≤ 4,000 × g, the microbial community sediments are relatively homogeneous. Under low-speed conditions, bacterial sedimentation appears to be driven primarily by bulk physical properties rather than by cell traits (e.g., size or density) (Iammarino et al., 2007). Consequently, the pellet collected under these conditions represents a nonselective sample of the microbiota. This observation was different from that of the high-speed centrifugation group. Significant differences in the diversity and selective enrichment of specific taxa were detected between the supernatant and the pellet. This result indicated that high-speed centrifugation preferentially sediments certain taxa (e.g., some Firmicutes) while retaining others (e.g., certain Proteobacteria) in the supernatant. The central implication is that low-to-medium centrifugal forces (500–4,000 × g in the present study) may offer a balanced compromise between bacterial recovery efficiency and preservation of community fidelity.

The differential partitioning of microbial taxa between the supernatant and pellet also underscores the issue of cell loss during centrifugation. Although increasing the rotational speed progressively reduced the OD_600_ of the supernatant and differential centrifugation visibly clarified the supernatant at higher g-forces, this loss could only be mitigated and not eliminated. At all the tested speeds, the supernatant OD_600_ remained greater than 0.05, indicating continuous bacterial loss. Thus, the discarded supernatant always contained a nonnegligible fraction of microbes regardless of the centrifugal force.

Although no significant differences in global α-diversity or β-diversity were detected among the treatment groups, the relative abundance of specific genera (e.g., *Delftia* and *Acinetobacter*) varied with processing method. Previous work has shown that elevated *Delftia* abundance coincides with the activation of phenylalanine metabolism pathways, suggesting a role in host amino acid processing or the generation of metabolites such as phenolic compounds (Fan et al., 2025b). Functional prediction with PICRUSt further revealed that while KEGG metabolic pathways were not significantly altered, several COG functional categories (e.g., COG2189, COG5008, and COG5022) differed among the groups, implying that preparation methods can modulate the functional gene expression potential of the microbiota even when the community structure remains unchanged. This observation broadens our understanding of FMT processing: even when the taxonomic composition is similar, the functional state of the microbiota may differ according to the processing protocol, thereby influencing post-transplantation engraftment and therapeutic efficacy. For example, the predicted enrichment of maltooligosyl trehalose synthase genes (COG3280) suggests it could theoretically influence carbohydrate availability and indirectly affect butyrate production, potentially influencing intestinal energy homeostasis (den Besten et al., 2013; Mayorga-Ramos et al., 2022; El-Salhy et al., 2021). However, without metabolomic validation, we cannot confirm actual enzymatic activity. This, to some extent, explains the inconsistent outcomes of FMT trials for complex disorders such as inflammatory bowel disease and irritable bowel syndrome across different centers.

When differential centrifugation was compared with single-step centrifugation at the same maximum g-force, no significant difference in microbial viability was observed. However, the amount of debris was markedly reduced. This finding indicates that differential centrifugation can decrease impurities without increasing mechanical injury. As the disappearance of the dark brown layer visible to the naked eye after differential centrifugation, this may represent a reduction in debris. Laboratory-based protocols for fecal microbiota preparation are recognized risk factors for FMT-related adverse events, and a decrease in debris content can significantly lower the incidence of such events in patients receiving FMT (Ding et al., 2019).

The strengths of this study include the use of large-volume human fecal samples, integrated viability and sequencing analyses, and a comprehensive evaluation of both single-step and differential centrifugation protocols. These features increase the clinical relevance of our findings, since FMT procedures involving human feces in routine practice often require the processing of substantial fecal masses (Yu et al., 2023). Limitations include the small donor cohort, the absence of an anaerobic workstation, and the lack of *in vivo* validation of transplant efficacy. CFU enumeration validated LIVE/DEAD staining trends. We acknowledge that aerobic BHI plating captures aerotolerant, cultivable fractions. While it is not suitable for strict anaerobes and fastidious organisms. Future studies should validate these findings using anaerobic cultivation conditions to specifically assess the impact on obligate anaerobes. We acknowledge that cell clumping or aggregation may affect accurate quantification. To minimize this, samples were thoroughly vortexed before staining, and five random fields were analyzed per sample. However, small aggregates may still be present, potentially leading to underestimation of cell counts. The use of V3-V4 region sequencing rather than whole-metagenome sequencing, which may limit taxonomic resolution. PICRUSt analysis identifies only predicted functional gene content derived from 16S rDNA data. The absence of accompanying proteomic or metabolomic data means we cannot confirm whether these predicted COG differences translate to altered enzymatic activities or metabolic fluxes. Future studies validate these *in vitro* observations in clinical FMT settings or *in vivo* animal models. For example, comparative transplantation experiments using murine colitis models. Study should assess whether filtration-preserved microbiota demonstrates superior engraftment and therapeutic efficacy compared to centrifugation-concentrated preparations. Previous animal work by Keller et al. reported a low cure rate after repeated centrifugation and washing of the fecal microbiota, implying that centrifugation can compromise the microbiota (Keller et al., 2021). He et al. compared transplantation of whole fecal suspensions (containing both bacteria and supernatant) with supernatant-only transplantation and concluded that the former yielded superior efficacy (He et al., 2019). Our data provide a mechanistic framework that may explain these observations. However, a clinical study by Wang et al. revealed that repeated centrifugation and resuspension washing did not significantly reduce the short-term clinical efficacy of FMT (Wang et al., 2018). The study noted a downward trend in efficacy despite the lack of statistical significance.

## 5 Conclusion

A comprehensive assessment demonstrated that, compared with any centrifugation protocol, filtration alone results in a fecal microbiota suspension that more closely resembles fresh feces. Regardless of the centrifugation scheme or speed, centrifugation reduces microbial viability and alters community structure and function prediction. Differential centrifugation, however, removes impurities more effectively than single-step centrifugation does. The selection of a fecal suspension preparation protocol should therefore be made cautiously, with comprehensive consideration of the abovementioned factors according to the intended research or clinical objective.

## Data Availability

The raw sequencing data presented in the study are deposited in the SRA, accession number PRJNA1317642.

## References

[ref1] Agus A. Clément K. Sokol H. (2021). Gut microbiota-derived metabolites as central regulators in metabolic disorders. Gut 70, 1174–1182. doi: 10.1136/gutjnl-2020-323071, 33272977 PMC8108286

[ref2] Basson A. R. Zhou Y. Seo B. Rodriguez-Palacios A. Cominelli F. (2020). Autologous fecal microbiota transplantation for the treatment of inflammatory bowel disease. Transl. Res. 226, 1–11. doi: 10.1016/j.trsl.2020.05.008, 32585148 PMC7308243

[ref3] Bellali S. Lagier J. C. Raoult D. Bou Khalil J. (2019). Among live and dead Bacteria, the optimization of sample collection and processing remains essential in recovering gut microbiota components. Front. Microbiol. 10:1606. doi: 10.3389/fmicb.2019.01606, 31354688 PMC6635563

[ref4] Bénard M. V. Arretxe I. Wortelboer K. Harmsen H. J. M. Davids M. De Bruijn C. M. A. . (2023). Anaerobic feces processing for fecal microbiota transplantation improves viability of obligate anaerobes. Microorganisms 11:11. doi: 10.3390/microorganisms11092238, 37764082 PMC10535047

[ref5] Borody T. J. Khoruts A. (2011). Fecal microbiota transplantation and emerging applications. Nat. Rev. Gastroenterol. Hepatol. 9, 88–96. doi: 10.1038/nrgastro.2011.244, 22183182

[ref7] Caporaso J. G. Kuczynski J. Stombaugh J. Bittinger K. Bushman F. D. Costello E. K. . (2010). Qiime allows analysis of high-throughput community sequencing data. Nat. Methods 7, 335–336. doi: 10.1038/nmeth.f.303, 20383131 PMC3156573

[ref8] Chen H. Boutros P. C. (2011). VennDiagram: a package for the generation of highly-customizable Venn and Euler diagrams in R. BMC Bioinformatics 12:35. doi: 10.1186/1471-2105-12-35, 21269502 PMC3041657

[ref9] Cole J. R. Wang Q. Fish J. A. Chai B. Mcgarrell D. M. Sun Y. . (2014). Ribosomal database project: data and tools for high throughput rRNA analysis. Nucleic Acids Res. 42, D633–D642. doi: 10.1093/nar/gkt1244 24288368 PMC3965039

[ref10] Cui B. Xu F. Zhang F. (2016). Methodology, not concept of fecal microbiota transplantation, affects clinical findings. Gastroenterology 150, 285–286. doi: 10.1053/j.gastro.2015.05.065, 26616573

[ref11] Danne C. Rolhion N. Sokol H. (2021). Recipient factors in faecal microbiota transplantation: one stool does not fit all. Nat. Rev. Gastroenterol. Hepatol. 18, 503–513. doi: 10.1038/s41575-021-00441-5 33907321

[ref6] De Cáceres M. Legendre P. (2009). Associations between species and groups of sites: indices and statistical inference. Ecology 90, 3566–3574. doi: 10.1890/08-1823.1 20120823

[ref12] Den Besten G. Van Eunen K. Groen A. K. Venema K. Reijngoud D. J. Bakker B. M. (2013). The role of short-chain fatty acids in the interplay between diet, gut microbiota, and host energy metabolism. J. Lipid Res. 54, 2325–2340. doi: 10.1194/jlr.R036012, 23821742 PMC3735932

[ref13] Desantis T. Z. Hugenholtz P. Larsen N. Rojas M. Brodie E. L. Keller K. . (2006). Greengenes, a chimera-checked 16S rrna gene database and workbench compatible with arb. Appl. Environ. Microbiol. 72, 5069–5072. doi: 10.1128/AEM.03006-05, 16820507 PMC1489311

[ref14] Ding X. Li Q. Li P. Zhang T. Cui B. Ji G. . (2019). Long-term safety and efficacy of fecal microbiota transplant in active ulcerative colitis. Drug Saf. 42, 869–880. doi: 10.1007/s40264-019-00809-2, 30972640

[ref15] El-Salhy M. Valeur J. Hausken T. Gunnar Hatlebakk J. (2021). Changes in fecal short-chain fatty acids following fecal microbiota transplantation in patients with irritable bowel syndrome. Neurogastroenterol. Motil. 33:e13983. doi: 10.1111/nmo.13983, 32945066 PMC7900992

[ref16] Fan L. Chen J. Zhang Q. Ren J. Chen Y. Yang J. . (2025). Fecal microbiota transplantation for hypertension: an exploratory, multicenter, randomized, blinded, placebo-controlled trial. Microbiome 13:133. doi: 10.1186/s40168-025-02118-6 40410854 PMC12100813

[ref17] Fan Y. Dong Y. Gai Z. Zhang Y. Han M. (2025). Lactiplantibacillus plantarum Lp05 protects against ethanol-induced liver injury in zebrafish through metabolic and microbiota modulation. Sci. Rep. 15:22584. doi: 10.1038/s41598-025-07111-5 40593078 PMC12216972

[ref18] Gasol J. Morán X. A. (1999). Effects of filtration on bacterial activity and picoplankton community structure as assessed by flow cytometry. Aquat. Microb. Ecol. 16, 251–264. doi: 10.3354/ame016251

[ref19] Hamilton M. J. Weingarden A. R. Sadowsky M. J. Khoruts A. (2012). Standardized frozen preparation for transplantation of fecal microbiota for recurrent *Clostridium difficile* infection. Am. J. Gastroenterol. 107, 761–767. doi: 10.1038/ajg.2011.482, 22290405

[ref20] He Y. Li X. Yu H. Ge Y. Liu Y. Qin X. . (2019). The functional role of fecal microbiota transplantation on dextran sulfate sodium-induced colitis in mice. Front. Cell. Infect. Microbiol. 9:393. doi: 10.3389/fcimb.2019.00393, 31803633 PMC6873233

[ref21] Hu J. Chen L. Tang Y. Xie C. Xu B. Shi M. . (2018). Standardized preparation for fecal microbiota transplantation in pigs. Front. Microbiol. 9:1328. doi: 10.3389/fmicb.2018.01328, 29971061 PMC6018536

[ref22] Iammarino M. J. Nti-Gyabaah J. Chandler M. D. Roush D. W. Göklen K. E. (2007). *Impact of Cell Density and Viability on Primary Clarification of Mammalian Cell Broth an Analysis Using Disc-Stack Centrifugation and Charged Depth Filtration*.

[ref23] Karimi M. Shirsalimi N. Hashempour Z. Salehi Omran H. Sedighi E. Beigi F. . (2024). Safety and efficacy of fecal microbiota transplantation (Fmt) as a modern adjuvant therapy in various diseases and disorders: a comprehensive literature review. Front. Immunol. 15:1439176. doi: 10.3389/fimmu.2024.1439176, 39391303 PMC11464302

[ref24] Keller J. J. Ooijevaar R. E. Hvas C. L. Terveer E. M. Lieberknecht S. C. Högenauer C. . (2021). A standardised model for stool banking for faecal microbiota transplantation: a consensus report from a multidisciplinary Ueg working group. United European Gastroenterol J 9, 229–247. doi: 10.1177/2050640620967898, 33151137 PMC8259288

[ref25] Lai Z. L. Tseng C. H. Ho H. J. Cheung C. K. Y. Lin J. Y. Chen Y. J. . (2018). Fecal microbiota transplantation confers beneficial metabolic effects of diet and exercise on diet-induced obese mice. Sci. Rep. 8:15625. doi: 10.1038/s41598-018-33893-y 30353027 PMC6199268

[ref26] Langille M. G. Zaneveld J. Caporaso J. G. Mcdonald D. Knights D. Reyes J. A. . (2013). Predictive functional profiling of microbial communities using 16S rrna marker gene sequences. Nat. Biotechnol. 31, 814–821. doi: 10.1038/nbt.2676, 23975157 PMC3819121

[ref27] Lin D. M. Koskella B. Ritz N. L. Lin D. Carroll-Portillo A. Lin H. C. (2019). Transplanting fecal virus-like particles reduces high-fat diet-induced small intestinal bacterial overgrowth in mice. Front. Cell. Infect. Microbiol. 9:348. doi: 10.3389/fcimb.2019.00348 31750259 PMC6843071

[ref28] Liu J. Miyake H. Zhu H. Li B. Alganabi M. Lee C. . (2020). Fecal microbiota transplantation by enema reduces intestinal injury in experimental necrotizing enterocolitis. J. Pediatr. Surg. 55, 1094–1098. doi: 10.1016/j.jpedsurg.2020.02.035, 32234317

[ref29] Magoč T. Salzberg S. L. (2011). Flash: fast length adjustment of short reads to improve genome assemblies. Bioinformatics 27, 2957–2963. doi: 10.1093/bioinformatics/btr507, 21903629 PMC3198573

[ref30] Martin M. (2011). Cutadapt removes adapter sequences from high-throughput sequencing reads. Embnet Journal 17:200. doi: 10.14806/ej.17.1.200

[ref31] Mayorga-Ramos A. Barba-Ostria C. Simancas-Racines D. Guamán L. P. (2022). Protective role of butyrate in obesity and diabetes: new insights. Front. Nutr. 9:1067647. doi: 10.3389/fnut.2022.1067647, 36505262 PMC9730524

[ref32] Mi F. Wang X. Zheng W. Wang J. Lin T. Sun M. . (2023). Effects of different preparation methods on microbiota composition of fecal suspension. Mol. Biotechnol. 65, 871–880. doi: 10.1007/s12033-022-00590-1, 36315335

[ref33] Mingaila J. Atzeni A. Burokas A. (2023). A comparison of methods of gut microbiota transplantation for preclinical studies. Int. J. Mol. Sci. 24:2005. doi: 10.3390/ijms241512005 37569381 PMC10418867

[ref34] Nguyen T. L. Vieira-Silva S. Liston A. Raes J. (2015). How informative is the mouse for human gut microbiota research? Dis. Model. Mech. 8, 1–16. doi: 10.1242/dmm.017400, 25561744 PMC4283646

[ref35] Nicco C. Paule A. Konturek P. Edeas M. (2020). From donor to patient: collection, preparation and cryopreservation of fecal samples for fecal microbiota transplantation. Diseases 8:9. doi: 10.3390/diseases8020009, 32326509 PMC7349373

[ref36] Oksanen J. Blanchet F. G. Kindt R. Legendre P. Minchin P. R. O’hara R. B. . (2013). Vegan: community ecology package. R package version. 2.0-10. doi: 10.32614/CRAN.package.vegan

[ref37] Ott S. J. Musfeldt M. Timmis K. N. Hampe J. Wenderoth D. F. Schreiber S. (2004). In vitro alterations of intestinal bacterial microbiota in fecal samples during storage. Diagn. Microbiol. Infect. Dis. 50, 237–245. doi: 10.1016/j.diagmicrobio.2004.08.012 15582296

[ref38] Paulson J. N. Stine O. C. Bravo H. C. Pop M. (2013). Differential abundance analysis for microbial marker-gene surveys. Nat. Methods 10, 1200–1202. doi: 10.1038/nmeth.2658 24076764 PMC4010126

[ref39] Perez E. Lee C. H. Petrof E. O. (2016). A practical method for preparation of fecal microbiota transplantation. Methods Mol. Biol. 1476, 259–267. doi: 10.1007/978-1-4939-6361-4_19 27507347

[ref40] Peterson B. W. Sharma P. K. Van Der Mei H. C. Busscher H. J. (2012). Bacterial cell surface damage due to centrifugal compaction. Appl. Environ. Microbiol. 78, 120–125. doi: 10.1128/AEM.06780-11, 22038609 PMC3255633

[ref41] Price M. N. Dehal P. S. Arkin A. P. (2009). FastTree: computing large minimum evolution trees with profiles instead of a distance matrix. Mol. Biol. Evol. 26, 1641–1650. doi: 10.1093/molbev/msp077 19377059 PMC2693737

[ref42] Proença I. M. Allegretti J. R. Bernardo W. M. De Moura D. T. H. Ponte Neto A. M. Matsubayashi C. O. . (2020). Fecal microbiota transplantation improves metabolic syndrome parameters: systematic review with meta-analysis based on randomized clinical trials. Nutr. Res. 83, 1–14. doi: 10.1016/j.nutres.2020.06.018 32987284

[ref43] Quaranta G. Guarnaccia A. Fancello G. Agrillo C. Iannarelli F. Sanguinetti M. . (2022). Fecal microbiota transplantation and other gut microbiota manipulation strategies. Microorganisms 10:2424. doi: 10.3390/microorganisms10122424, 36557677 PMC9781458

[ref44] Randolph N. K. Salerno M. Klein H. Diaz-Campos D. Van Balen J. C. Winston J. A. (2025). Preparation of fecal microbiota transplantation products for companion animals. Public Libr. Sci. One 20:e0319161. doi: 10.1371/journal.pone.0319161 PMC11981653 40203217

[ref45] Rokkas T. Gisbert J. P. Gasbarrini A. Hold G. L. Tilg H. Malfertheiner P. . (2019). A network meta-analysis of randomized controlled trials exploring the role of fecal microbiota transplantation in recurrent *Clostridium difficile* infection. United European Gastroenterol J 7, 1051–1063. doi: 10.1177/2050640619854587 PMC6794697 31662862

[ref46] Secombe K. R. Al-Qadami G. H. Subramaniam C. B. Bowen J. M. Scott J. Van Sebille Y. Z. A. . (2021). Guidelines for reporting on animal fecal transplantation (graft) studies: recommendations from a systematic review of murine transplantation protocols. Gut Microbes 13:1979878. doi: 10.1080/19490976.2021.1979878 34586011 PMC8489962

[ref47] Sedeek S. A. Farowski F. Youssafi S. Tsakmaklis A. Brodesser S. El-Attar M. M. . (2025). In vitro validation concept for lyophilized fecal microbiota products with a focus on bacterial viability. World J. Microbiol. Biotechnol. 41:83. doi: 10.1007/s11274-025-04291-0 40011318 PMC11865215

[ref48] Stebegg M. Silva-Cayetano A. Innocentin S. Jenkins T. P. Cantacessi C. Gilbert C. . (2019). Heterochronic faecal transplantation boosts gut germinal centres in aged mice. Nat. Commun. 10:2443. doi: 10.1038/s41467-019-10430-7, 31164642 PMC6547660

[ref49] Tuddenham S. Sears C. L. (2015). The intestinal microbiome and health. Curr. Opin. Infect. Dis. 28, 464–470. doi: 10.1097/QCO.0000000000000196, 26237547 PMC4643846

[ref50] Waller K. M. J. Leong R. W. Paramsothy S. (2022). An update on fecal microbiota transplantation for the treatment of gastrointestinal diseases. J. Gastroenterol. Hepatol. 37, 246–255. doi: 10.1111/jgh.15731 34735024

[ref51] Wang H. Cui B. Li Q. Ding X. Li P. Zhang T. . (2018). The safety of fecal microbiota transplantation for Crohn's disease: findings from a long-term study. Adv. Ther. 35, 1935–1944. doi: 10.1007/s12325-018-0800-3, 30328062 PMC6223988

[ref52] Wickham H. (2016). ggplot2: Elegant Graphics for Data Analysis. Berlin: Springer Publishing Company.

[ref53] Wu Z. Huang S. Li T. Li N. Han D. Zhang B. . (2021). Gut microbiota from green tea polyphenol-dosed mice improves intestinal epithelial homeostasis and ameliorates experimental colitis. Microbiome 9:184. doi: 10.1186/s40168-021-01115-9 34493333 PMC8424887

[ref54] Yadegar A. Bar-Yoseph H. Monaghan T. M. Pakpour S. Severino A. Kuijper E. J. . (2024). Fecal microbiota transplantation: current challenges and future landscapes. Clin. Microbiol. Rev. 37:e0006022. doi: 10.1128/cmr.00060-22, 38717124 PMC11325845

[ref55] Yang L. Hou K. Zhang B. Ouyang C. Lin A. Xu S. . (2020). Preservation of the fecal samples at ambient temperature for microbiota analysis with a cost-effective and reliable stabilizer EffcGut. Sci. Total Environ. 741:140423. doi: 10.1016/j.scitotenv.2020.140423 32615432

[ref56] Yu Y. Wang W. Zhang F. (2023). The next generation fecal microbiota transplantation: to transplant Bacteria or Virome. Adv. Sci. 10:e2301097. doi: 10.1002/advs.202301097, 37914662 PMC10724401

[ref57] Zain N. M. M. Ter Linden D. Lilley A. K. Royall P. G. Tsoka S. Bruce K. D. . (2022). Design and manufacture of a lyophilised faecal microbiota capsule formulation to Gmp standards. J. Control. Release 350, 324–331. doi: 10.1016/j.jconrel.2022.08.012 35963468

[ref58] Zhang X. Y. Chen Q. Y. Li N. Qin H. L. (2020). Indication selection and clinical application strategies of fecal microbiota transplantation. Chin. J. Gastrointes. Surg. 23, 509–515. doi: 10.3760/cma.j.cn.441530-20200110-00015, 32842434

[ref59] Zhang X. Luo X. Tian L. Yue P. Li M. Liu K. . (2023). The gut microbiome dysbiosis and regulation by fecal microbiota transplantation: umbrella review. Front. Microbiol. 14:1286429. doi: 10.3389/fmicb.2023.1286429 38029189 PMC10655098

[ref60] Zhao H. L. Chen S. Z. Xu H. M. Zhou Y. L. He J. Huang H. L. . (2020). Efficacy and safety of fecal microbiota transplantation for treating patients with ulcerative colitis: a systematic review and meta-analysis. J. Dig. Dis. 21, 534–548. doi: 10.1111/1751-2980.12933, 33439534

[ref61] Zhao Z. Ning J. Bao X. Q. Shang M. Ma J. Li G. . (2021). Fecal microbiota transplantation protects rotenone-induced Parkinson's disease mice via suppressing inflammation mediated by the lipopolysaccharide-Tlr4 signaling pathway through the microbiota-gut-brain axis. Microbiome 9:226. doi: 10.1186/s40168-021-01107-9, 34784980 PMC8597301

[ref62] Zhou J. Zhou Z. Ji P. Ma M. Guo J. Jiang S. (2019). Effect of fecal microbiota transplantation on experimental colitis in mice. Exp. Ther. Med. 17, 2581–2586. doi: 10.3892/etm.2019.7263, 30906449 PMC6425147

