# Supplementary material for: The effects of filtration and centrifugation on the gut microbiota in fecal microbiota transplantation preparation
Source: Front Microbiol. 2026 May 12;17:1768356. doi: 10.3389/fmicb.2026.1768356 (PMC13201525; doi:10.3389/fmicb.2026.1768356)
Supplement: Supplementary file 1 [file Supplementary_File_1.DOCX]

| No. | 1 | 2 | 3 | 4 | 5 | 6 | 7 | 8 | 9 |
| --- | --- | --- | --- | --- | --- | --- | --- | --- | --- |
| Sex | Male | Female | Female | Male | Male | Female | Female | Female | Female |
| Age（years） | 60 | 33 | 25 | 25 | 26 | 27 | 30 | 29 | 26 |
| Ethnicity | Han | Han | Han | Han | Han | Han | Han | Han | Han |
| Medical history | None | None | None | None | None | None | None | None | None |
| Antibiotic use in past 3 months | No | No | No | No | No | No | No | No | No |

## Table S1 Clinical characteristics of the fecal donors.


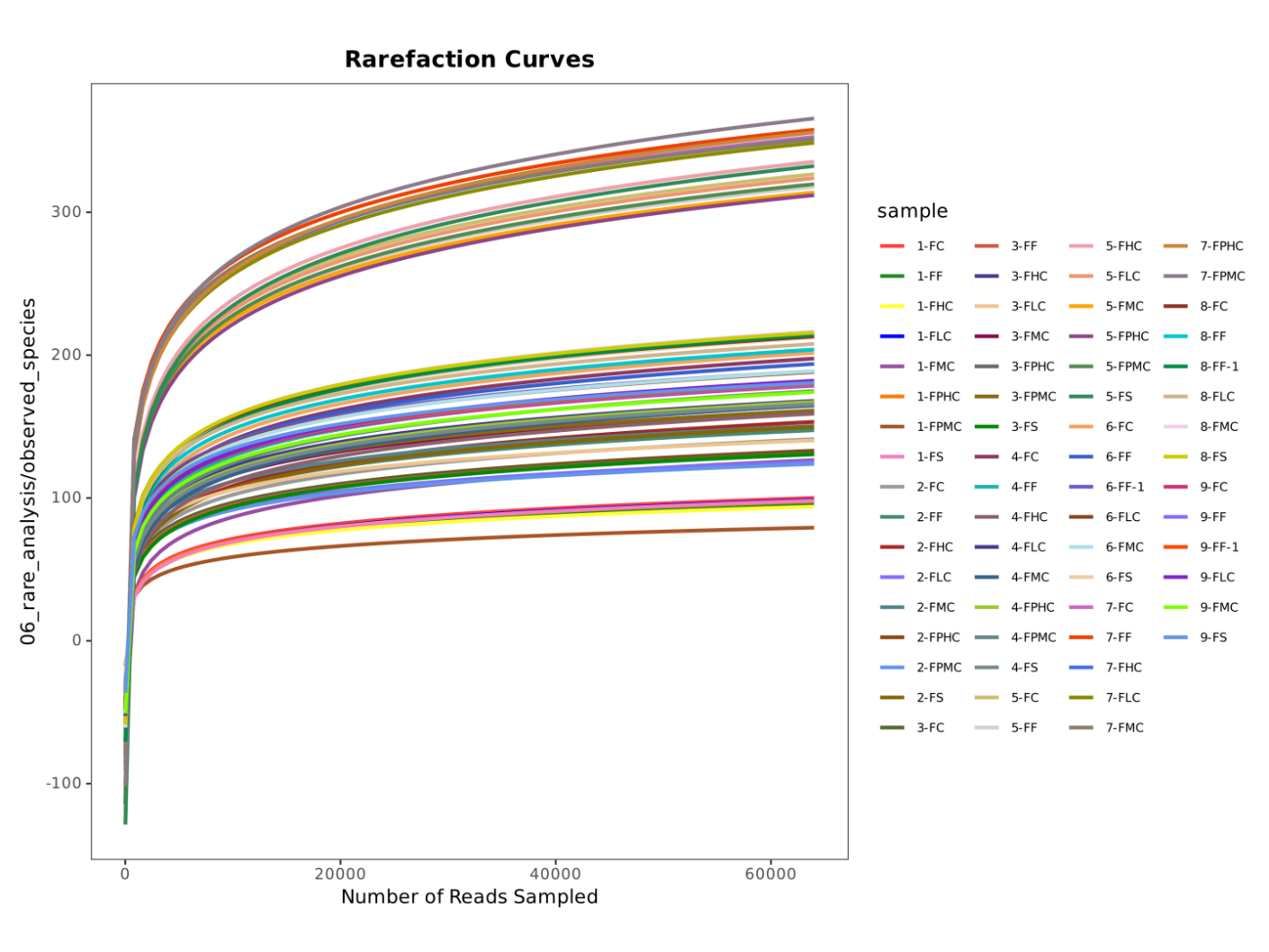


**Figure S1 Rarefaction curves of the observed species in all the samples.** The stabilization of the rarefaction curves derived from the sequencing data indicates adequate sampling coverage at this sequencing.


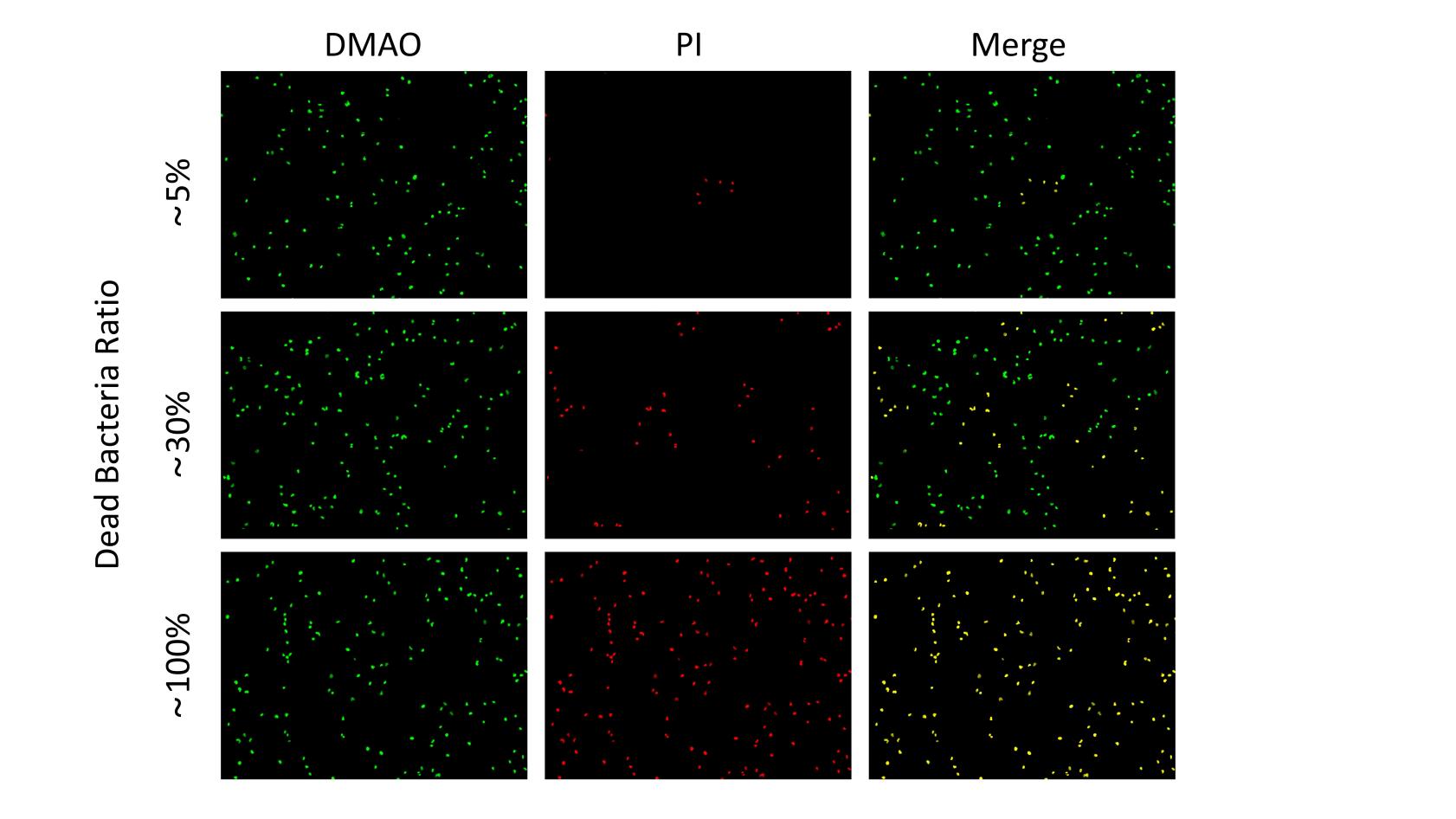


****Figure S2 Validation of DMAO/PI staining specificity using *E. coli* viability controls.** The left column shows DMAO fluorescence (green; labeling all bacteria with intact or compromised membranes). The middle column shows PI fluorescence (red; labeling only membrane-compromised/dead bacteria). The right column shows merged images. Top row: live *E. coli* control (~5% dead bacteria ratio), exhibiting predominantly green fluorescence with minimal PI signal. Middle row: mixed viability population (~30% dead bacteria ratio), showing both green (live) and yellow/orange (dead) signals. Bottom row: heat-killed *E. coli* control (~100% dead bacteria ratio), exhibiting predominant red/yellow fluorescence due to extensive membrane damage.**


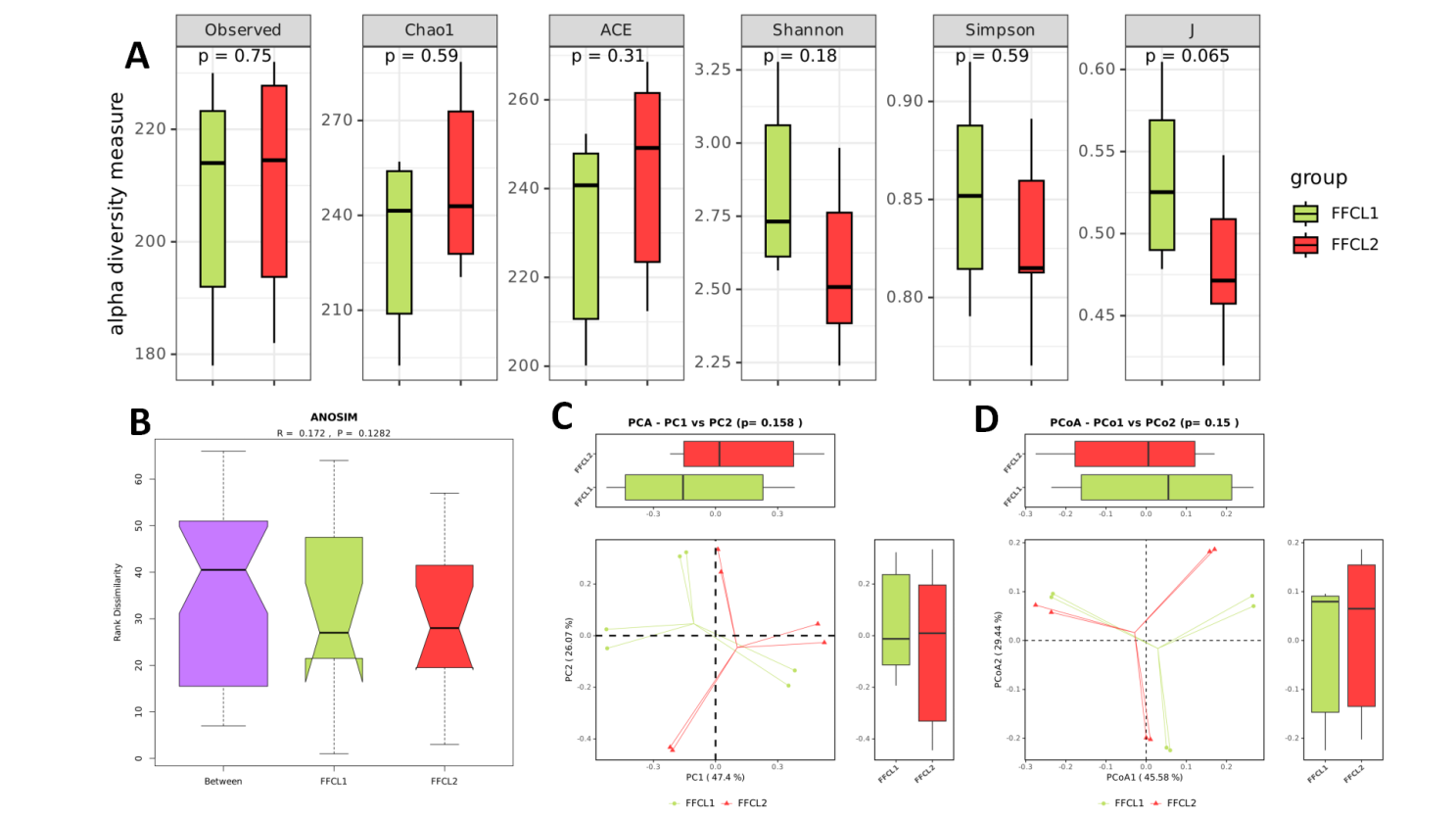


**Figure S3 Comparison of microbial community alpha and beta diversity between the FFCL1 and FFCL2 groups. (A).** Comparison of different alpha diversity indices (observed, Chao1, ACE, Shannon, Simpson, J) between the two groups, with no significant differences observed. **(B).** ANOSIM results between the two groups, with R = 0.172 and p = 0.1282, indicating no significant differences.**(C).** and **(D).** show the results of PCA and PCoA-based principal component analysis, which revealed no significant distribution differences between FFCL1 and FFCL2 (p = 0.158 and p = 0.15).


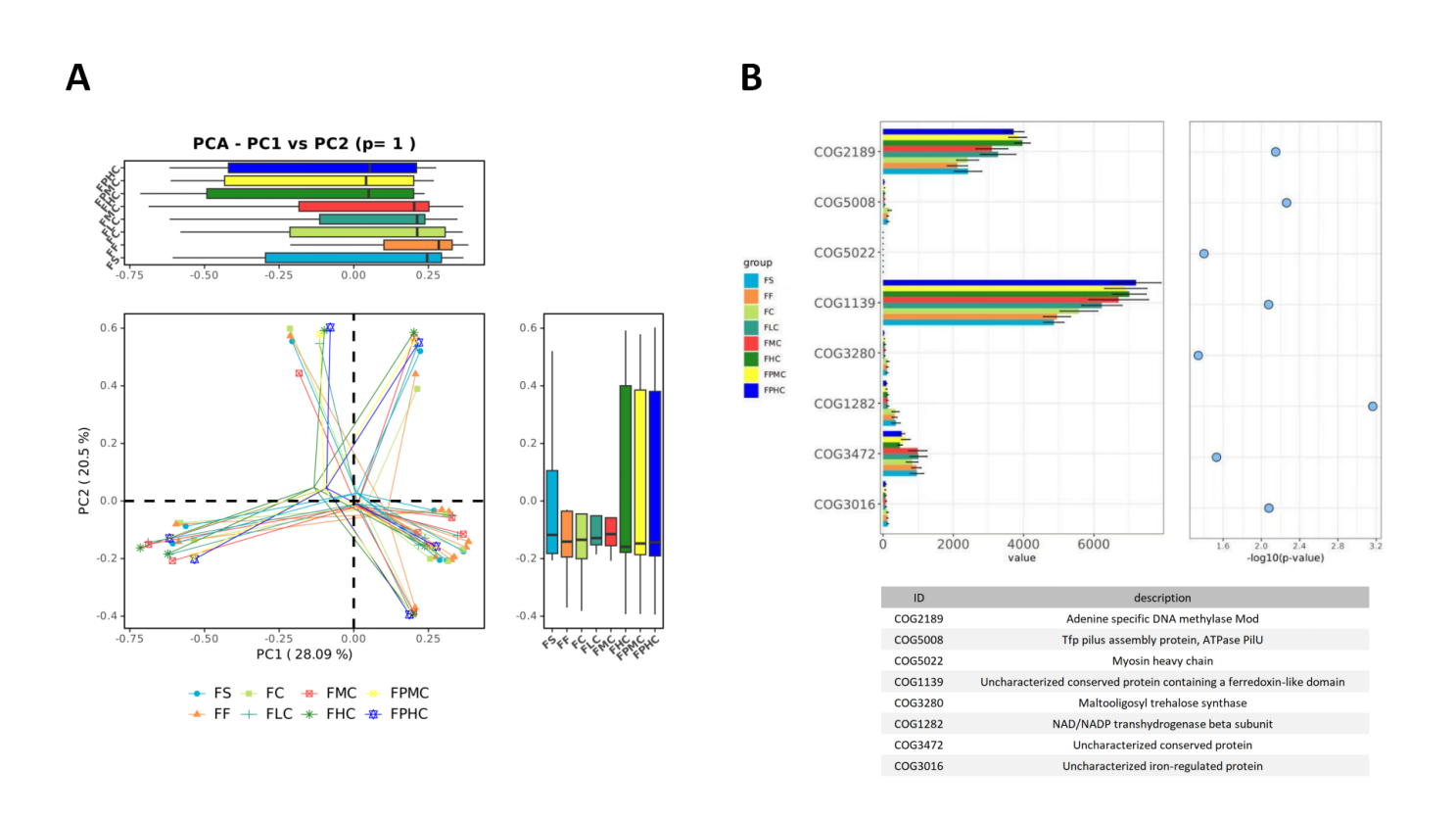


**Figure S4 Microbial community composition and beta diversity in different treatment groups. (A)**. The results of PCA-based principal component analysis, demonstrating no significant distribution differences among the treatment groups in PC1 and PC2 (p = 1). **(B)**. Comparison of COG-based functional profiles of fecal microbial communities between different treatment groups.
